# Photoperiodic Regulation of SK Channels in Dorsal Raphe Serotonin Neurons

**DOI:** 10.1111/ejn.70752

**Published:** 2026-11-05

**Authors:** Manuel A. Giannoni‐Guzmán, José C. Zepeda, Anna Kamitakahara, Valerie Magalong, Pat Levitt, Brad A. Grueter, Douglas G. McMahon

**Affiliations:** ^1^ Department of Biological Sciences Vanderbilt University Nashville Tennessee USA; ^2^ Department of Biology Middle Tennessee State University Murfreesboro Tennessee USA; ^3^ Department of Pharmacology Vanderbilt University Nashville Tennessee USA; ^4^ Department of Pediatrics and Program in Developmental Neuroscience and Neurogenetics, The Saban Research Institute, Children's Hospital Los Angeles, Keck School of Medicine University of Southern California Los Angeles California USA; ^5^ Department of Anesthesiology Vanderbilt University School of Medicine Nashville Tennessee USA; ^6^ Vanderbilt Brain Institute Vanderbilt University School of Medicine Nashville Tennessee USA; ^7^ Vanderbilt Center for Addiction Research Vanderbilt University School of Medicine Nashville Tennessee USA

**Keywords:** dorsal raphe nucleus, electrophysiology, melatonin signaling, photoperiod, serotonin, SK channel

## Abstract

Day length, or seasonal photoperiod, shapes mood and affective behaviors, but the neural mechanisms underlying these effects are still being defined. Serotonin neurons of the dorsal raphe nucleus (DRN) are critical regulators of affective behaviors, and photoperiod modulates their excitability and ongoing activity. Here, we investigated the influence of seasonal photoperiod on the function and expression of small conductance calcium‐activated potassium (SK) channels, which mediate the after‐hyperpolarizing potential (AHP) in dorsal raphe serotonin neurons. Building on previous work demonstrating that long summer‐like photoperiod increases serotonergic neuron excitability and decreases AHP amplitude, we hypothesized that day length influences SK channel activity, thereby contributing to differences in neuronal excitability observed between Long, Equinox, and Short photoperiod conditions. Using multielectrode array recordings from DRN slices, we found a significant, dose‐dependent increase in spike rate upon application of the SK channel inhibitor apamin, indicating that SK channels indeed influence the spike rate of dorsal raphe serotonin neurons. In addition, whole‐cell recordings from DRN neurons in slices demonstrated that SK channel–mediated AHP currents were smaller in amplitude in Long photoperiod mice compared to Short and Equinox photoperiod mice. However, we found no significant differences in mRNA levels of the SK3 subunit (*Kcnn3*) in DRN serotonin neurons across photoperiod conditions as determined by single‐molecule fluorescence in situ hybridization. Overall, these findings indicate that photoperiod modulates SK channel function in DRN serotonin neurons, possibly through posttranscriptional mechanisms, contributing to the increase in serotonin neuron excitability observed in long summer‐like photoperiods.

## Key Points

- Seasonal changes in day length (photoperiod) influence mood and alter the activity of serotonin‐producing neurons in the brain.
- This study investigated how photoperiod affects SK channels, which contribute to the regulation of the firing rate of serotonin neurons in the dorsal raphe nucleus.
- Blocking SK channels increased neuronal firing rate, indicating that these channels reduce activity in serotonin neurons.
- SK channel currents were reduced in mice exposed to long day photoperiods, even though SK3 subunit (*Kcnn3*) gene expression levels remained the same.
- These results suggest that day length alters serotonin neuron activity, likely by modifying SK channel function, helping explain how seasons affect mood.

## Graphical abstract

Long summer‐like photoperiod reduces SK channel function in dorsal raphe serotonin neurons. SK channel blockade with apamin increases firing rate in slices from short‐photoperiod mice, mimicking the elevated firing seen in long‐photoperiod mice. Whole‐cell recordings show that apamin‐sensitive tail currents are smaller in long‐photoperiod neurons, while *Kcnn3* (SK3) mRNA in serotonin neurons is unchanged, pointing to posttranscriptional regulation of SK channels by day length.

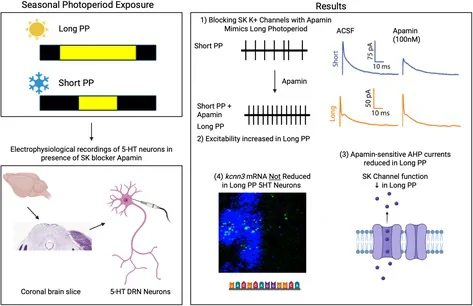

**Abbreviations**
5‐HT: 5‐hydroxytryptamine (serotonin)
aCSF: artificial cerebrospinal fluid
AHP: afterhyperpolarization
Aq: cerebral aqueduct
ANOVA: analysis of variance
CK2: casein kinase 2
CV: coefficient of variation
DRN: dorsal raphe nucleus
EPSP: excitatory postsynaptic potential
EGTA: ethylene glycol‐bis(β‐aminoethyl ether)‐N,N,N′,N′‐tetraacetic acid
Eqx: Equinox photoperiod (12:12 h LD)
HEPES: 4‐(2‐hydroxyethyl)‐1‐piperazineethanesulfonic acid
IACUC: Institutional Animal Care and Use Committee
IC50: half‐maximal inhibitory concentration
ISI: interspike interval
IPSP: inhibitory postsynaptic potential
Kcnk2: potassium two‐pore‐domain channel subfamily K member 2 (gene encoding TREK‐1)
*Kcnn3*
: potassium calcium‐activated channel subfamily N member 3 (gene encoding SK3)
LD: light:dark cycle
MEA: multielectrode array
MT1: melatonin receptor 1
NMDG: *N*‐methyl‐D‐glucamine
PKA: protein kinase A
PP2A: protein phosphatase 2A
RA: access resistance
ROI: region of interest
SCN: suprachiasmatic nucleus
SD: standard deviation
SK: small‐conductance Ca^2+^‐activated K^+^ channel
SK1, SK2, SK3: SK channel subunits 1, 2 and 3
TASK‐1: TWIK‐related acid‐sensitive K^+^ channel 1
*Tph2*
: tryptophan hydroxylase 2
TREK‐1: TWIK‐related K^+^ channel 1
5‐HT₁A: serotonin 1A receptor
8‐OH‐DPAT: 8‐hydroxy‐2‐(di‐*n*‐propylamino)tetralin

## 1 Introduction

Environmental signals sculpt brain development, resulting in changes in overall behavior and physiology (Kolb and Whishaw 1998; Meaney 2001). In mammals, early‐life experiences can have lasting effects on neural development, shaping behavioral phenotypes into adulthood (Andersen 2015; Boulle et al. 2016; Damulewicz et al. 2022; Pyter and Nelson 2006; Rincón‐Cortés and Sullivan 2014). Day length (photoperiod) is a key environmental cue that organisms use to synchronize their internal biological clocks with seasonal changes, thereby timing critical processes such as reproduction and development to maximize offspring survival (Goldman 2001; Nakane and Yoshimura 2019). Work in rodents has shown that exposure to seasonal photoperiods alters the function of serotonergic and dopaminergic neurons, potentially leading to changes in behavior (Green et al. 2015; Jameson et al. 2023, 2024; Kassaye et al. 2025; Pyter and Nelson 2006; Siemann et al. 2019, 2020). Research in human populations suggests associations between developmental photoperiod and lifetime risk for major depression (Devore et al. 2018; Lewis et al. 2024; Viejo‐Romero et al. 2024).

In mice, perinatal photoperiod exposure results in enduring changes to the master circadian clock located in the suprachiasmatic nucleus (SCN) (Ciarleglio et al. 2011; Cox et al. 2024). The dorsal raphe nucleus (DRN), the largest of the serotonergic nuclei in the brain (Hornung 2010), receives light input from both the master clock and the circadian visual system (Gooley et al. 2003; Morin 1999, 2013). Using the C3Hf+/+ mouse strain, which possesses intact melatonin signaling critical for mediating neuronal and behavioral responses to seasonal photoperiod, our group has shown that photoperiodic signals influence serotonergic brain circuits (Giannoni‐Guzmán et al. 2021; Green et al. 2015; Siemann et al. 2019). Behaviorally, mice raised under Long (16:8 h LD) (summer‐like) photoperiods present decreased depressive and anxiety‐like behaviors as adults when compared to mice raised under Short (8:16 h LD) (winter‐like) photoperiods (Green et al. 2015; Siemann et al. 2019). Serotonin neurons in DRN slices from Long mice exhibit increased excitability, characterized by depolarization of the resting membrane potential and a decreased afterhyperpolarization (AHP) amplitude (Green et al. 2015). We have shown that the transcriptional expression of the twin‐pore K + channel TREK‐1—a key ion channel setting the resting membrane potential and regulating neuronal excitability (Talley et al. 2001)—is significantly decreased in serotonin neurons from Long photoperiod mice compared to those from Short photoperiod mice (Giannoni‐Guzmán et al. 2021), likely contributing to their depolarized membrane potential. The behavioral, transcriptional and neural effects of photoperiodic programming were negated in melatonin receptor 1 (MT1) knockout mice, indicating that melatonin signaling is required for the changes induced by photoperiod in DRN serotonin neurons (Giannoni‐Guzmán et al. 2021; Green et al. 2015).

Significant gaps remain in our understanding of the intrinsic ion channel mechanisms that underlie photoperiodic programming of this system, such as the observed changes in the AHP of DRN serotonin neurons. In serotonin neurons of the DRN, the AHP is carried primarily by small conductance calcium‐activated K^+^ channels (SK) (Stocker 2004), with the SK3 channel presenting the highest expression in this brain region (Stocker and Pedarzani 2000). SK channel currents are selectively inhibited by the peptide toxin apamin (IC50 = 0.7–4 nM), which binds to the external pore region of the channel, thereby blocking potassium efflux and attenuating the AHP that regulates neuronal excitability (Crespi 2009; Grunnet et al. 2001; Kuzmenkov et al. 2022; van der Staay et al. 1999). Apamin has been shown to reduce depressive‐like behavior in rodents when administered systemically as well as modulating dorsal raphe serotonergic neuron firing, highlighting its potential to regulate affective behavior through SK channel inhibition (Sargin et al. 2016).

Here, we hypothesize that photoperiod modulates SK channel currents in DRN serotonergic neurons, leading to differential changes in the excitability and activity of DRN serotonin neurons between photoperiods. To test this hypothesis, we first determined how pharmacological inhibition of SK channels affects serotonin neuron activity under different photoperiod conditions using multielectrode array recordings. We then quantified SK currents in DRN serotonin neurons across photoperiods employing patch‐clamp electrophysiology. Lastly, we examined whether photoperiod alters *Kcnn3* (SK3) expression in DRN serotonin neurons using RNAscope.

## 2 Materials and Methods

### 2.1 Ethics Statement

All procedures involving animals in this study were reviewed and approved by the Vanderbilt University Institutional Animal Care and Use Committee (IACUC), under approval number D16‐00143 (A3227‐01). The experiments were conducted in strict accordance with the Guide for the Care and Use of Laboratory Animals published by the National Institutes of Health (NIH) and followed the national guidelines set forth by the United States Public Health Service (PHS). Animals had access to food and water ad libitum throughout the study. Because anesthetic agents are known to alter neuronal physiology in the DRN (McCardle and Gartside 2012) and the SCN (Chong et al. 2021), mice were euthanized via cervical dislocation without the use of anesthesia to avoid confounding effects. Every effort was made to minimize animal pain, distress, and discomfort as well as adherence to humane endpoints. The investigators affirm that we understand the ethical principles under which the journal operates and confirm that our work complies fully with the journal's animal ethics standards.

### 2.2 Animals and Housing

All mice used in our experiments were from the C3Hf+/+ mouse strain (a gift of Gianluca Tosini, Morehouse School of Medicine, Atlanta, GA). These mice (C3A.BLiA‐Pde6b+/J; JAX stock #001912) have intact melatonin signaling and do not carry the *rd* allele that causes retinal degeneration in the C3H/HeJ parent strain (Baba et al. 2009). Mice were paired and placed on either Long (16 h of light and 8 h of darkness), Equinox (12 h of light 12 h of darkness), or Short (8 h of light and 16 h of darkness) photoperiods. Litters were maintained in group housing under the same photoperiod until experiments were performed between postnatal days 40 and 60. First litters from breeders were not used. Tissue harvesting occurred between 11:00 a.m. and 12:00 p.m. local time, the mid‐day point of all light cycles. Tissue collection was performed at the midpoint of the light period to minimize potential variability due to circadian or time‐of‐day effects on gene expression and neuronal activity. All recordings were performed during the light phase. Male and female animals were used in equal numbers in each cohort.

### 2.3 Multielectrode Array Electrophysiological Recording

Mice were euthanized, brains were extracted and mounted in cold, oxygenated (95% O_2_–5%CO_2_) dissecting media (in mM: 114.5 NaCl, 3.5 KCl, 1 NaH_2_PO4, 1.3 MgSO_4_, 2.5 CaCl_2_, 10 D(+)‐glucose, and 35.7 NaHCO_3_), and 300‐μm thick coronal slices were taken using a Vibroslicer (Campden Instruments). Dorsal raphe nuclei were isolated by removing the extraneous cortical tissue and placed in a slice chamber full of room temperature, oxygenated, extracellular recording media (in mM: 124 NaCl, 3.5 KCl, 1 NaH2PO_4_, 1.3 MgSO_4_, 2.5 CaCl_2_,10 D(+)‐glucose, and 20 NaHCO3).

Multielectrode array recordings were performed by dissecting out the dorsal raphe nucleus after slicing, as previously described (Giannoni‐Guzmán et al. 2021; Manz et al. 2021). The tissue was then placed on a perforated electrode array and immobilized with a harp for recording. To elicit spontaneous firing of serotonin neurons, 40 μM tryptophan and 3 μM phenylephrine were added to the recording solution which was perfused (1.3 mL/s) over the slice, to ensure sufficient substrate for serotonin synthesis and to activate α₁‐adrenergic receptors that promote firing in otherwise quiescent dorsal raphe neurons in ex vivo conditions (Green et al. 2015; Wang et al. 2022). After 45 min of placing the slice in solution recording began. For SK channel inhibition, apamin (Tocris|Biotechne CAS No: 24345‐16‐2) was perfused (1.3 mL/s) over the slice in a serial manner at concentrations of 0, 1, 5, 50, and 100 nM. Each dose was perfused for 5 min to ensure the desired concentration in the bath. Lastly, the 5‐HT_1_A receptor agonist 8‐OH‐DPAT was perfused into the solution at 5 μM concentration to identify serotonergic cells by silencing. Single units were isolated by offline spike sorting (see Statistical Analysis). Firing rate for each unit was calculated as the number of spikes divided by the duration of each drug epoch (aCSF, ~300 s; each apamin concentration, ~300–400 s; 8‐OH‐DPAT, ~400 s), with epoch boundaries defined from the perfusion logs. Only units whose firing rate during 8‐OH‐DPAT fell below 50% of their rate in the preceding 100 nM apamin epoch were classified as serotonergic and included in the firing rate, ISI, and CV analyses; mean values were then computed per slice, and slices contributed one value each to the group statistics (Long *n* = 5, Equinox *n* = 6, Short *n* = 6). Recordings were included only if at least seven serotonergic units were identified per slice.

### 2.4 Whole‐Cell Patch‐Clamp Electrophysiology

Male and female mice were euthanized, their brains were removed, and 250 μm thick coronal brain slices were collected using a Leica VT1200S Vibratome. For all experiments, we focused our collection on the dorsal‐most 0.25‐mm subregion of the DRN to ensure consistency across experiments, as this region contains a dense population of serotonergic neurons. Slices were prepared in oxygenated (95% O_2_; 5%CO_2_) ice‐cold *N*‐methyl‐D‐glucamine (NMDG)‐based solution (in mM: 2.5 KCl, 20 HEPES, 1.2 NaH2PO4, 25 Glucose, 93 NMDG, 30 NaHCO3, 5.0 sodium ascorbate, 3.0 sodium pyruvate, 10 MgCl_2_, and 0.5 CaCl_2_‐2H_2_O) and recovered for 10 min in the same solution at 34°C. Slices were then recovered for 1 h in oxygenated artificial cerebrospinal fluid (aCSF) containing (in mM: 119 NaCl, 2.5 KCl, 1.3 MgCl_2_‐6H_2_O, 2.5 CaCl_2_‐2H_2_O, 1.0 NaH_2_PO_4_‐H_2_O, 26.2 NaHCO_3_, and 11 glucose; 287–295 mOsm). During experiments, slices were continuously perfused with oxygenated aCSF containing 3 μM phenylephrine to stimulate spontaneous action potentials at a rate of 2 mL/min and a temperature of 30°C–32°C. Whole‐cell patch‐clamp electrophysiology was performed using a CV‐7B head stage, Multiclamp 700B Amplifier, Axopatch Digidata 1550 digitizer, and 3–6 MΩ glass recording micropipettes (Sutter P1000 Micropipette Puller). Whole‐cell patch clamp recordings were performed using a K + ‐based intracellular solution (in mM: 135 + ‐gluconate, 5 NaCl, 2 MgCl_2_, 10 HEPES, 0.6 EGTA, 3 Na_2_ATP, 0.4 Na_2_GTP; 285–292 mOsm). Leak subtraction was not used, and the liquid junction potential was corrected. In order to identify serotonergic cells, spontaneous action potentials were recorded from neurons using the same intracellular solution before and during a bath application of the 5‐HT_1_A receptor agonist 8‐OH‐DPAT (5 μM). Only cells whose spontaneous action potentials decreased by > 50% in the presence of 8‐OH‐DPAT were included in this study. Spontaneous action potential frequency was measured in current clamp over a 3‐min window immediately before 8‐OH‐DPAT application and over a 3‐min window beginning 2 min after the start of 8‐OH‐DPAT perfusion, and the percentage change was calculated from these two values.

Neurons were held at −60 mV and subjected to a 2‐ms depolarizing voltage step to +30 mV, followed by repolarization to −60 mV to elicit outward tail currents. Tail‐current amplitude was quantified following repolarization by measuring the peak current after exclusion of the capacitive transient. Baseline current, calculated from the holding period preceding depolarization, was subtracted from each recording. Tail‐current amplitudes were measured from individual traces and averaged across repeated sweeps for each neuron. Tail current durations were defined as the time from repolarization to the return of the outward current to baseline. In one Equinox neuron, the tail current was too small (~2 pA) to define its duration; this cell was retained in the amplitude analyses but excluded from the duration analyses (Equinox *n* = 13 for duration). Inclusion criteria for cells were a stable (< 20% change) access resistance (RA) of < 20 MΩ, and a stable holding current when held at −70 mV.

For both the MEA recordings and the whole‐cell patch‐clamp experiments, phenylephrine (3 μM) was present continuously in the perfusate for all photoperiod groups and throughout all drug conditions, to supply the tonic α1‐adrenergic drive that dorsal raphe serotonin neurons receive in vivo from the locus coeruleus and that is required for sustained pacemaker firing in slices (Green et al. 2015; Vandermaelen and Aghajanian 1983; Wang et al. 2022). Apamin effects were therefore measured against a constant phenylephrine background within each cell or slice.

### 2.5 In Situ Hybridization Quantification

Whole brain tissue was collected (*n* = 6 per group) and submerged into isopentane for 25 s, placed in crushed dry ice for approximately 1 min, and stored in a 50 mL falcon tube at −80°C. Twenty‐micrometer sections were prepared per animal targeting the dorsal raphe nucleus from −5.5 to −5.75 mm from Bregma. Tissue sections were then processed by single‐molecule fluorescence in situ hybridization using RNAscope (Advanced Cell Diagnostics). Specific probes targeted the transcripts *Kcnn3* (SK3) and *Tph2* with fluorescent dyes Alexa488 and Atto647, respectively. Once tissue was processed, laser scans for each fluorescent channel containing the dorsal raphe nucleus for each brain slice were captured using a Zeiss LSM510 confocal scanning microscope (Carl Zeiss Microscopy Gmbh, Jena, Germany). Sixteen sequential scans at 40× of 512 × 512 pixels were taken by the microscope and stitched together to produce a high‐resolution composite of the area. Laser settings and pinhole sizes were the same for all the captures. Image analysis was performed using the open‐source software Fiji (https://imagej.net/Fiji). Colocalization of *Kcnn3* with *Tph2* was established using the plugin ComDet (https://github.com/ekatrukha/ComDet) (Fréal et al. 2019). Briefly, the plugin identifies small ROIs for each channel independently and assesses whether they colocalize spatially within up to 4 pixels of each other. The number of colocalized particles is reported as a proxy for gene expression levels. Four sections per animal were analyzed and averaged to yield a single value per animal; image acquisition and quantification were performed blind to photoperiod group, with identical settings across all groups.

### 2.6 Statistical Analysis

Data are presented as mean ± standard deviation, with individual data points shown when *n* ≤ 30. Multielectrode array traces were analyzed using offline sorting (Plexon) and spikes were sorted using a combination of manual identification and automatic k‐means‐based sorting software. Spike timestamps for each sorted unit were exported using the ‘Per‐Waveform Data’ option, which outputs the unit ID, timestamp, and waveform segment. The files were imported into Python, where timestamps were sorted chronologically, and ISIs were calculated as the difference between consecutive spike timestamps (ISI = *t*(*i* + 1) – *t*(*i*)). The coefficient of variation (CV) of ISIs was then calculated as the ratio of the standard deviation to the mean ISI (CV = *σ* (ISI)/*μ* (ISI)) to quantify the irregularity of spike timing within each treatment. The resulting datasets were used for subsequent statistical comparisons across experimental conditions and photoperiods.

Statistical analyses were performed in GraphPad Prism 10. For multielectrode array recordings (Figure 1), firing rate was analyzed by two‐way repeated‐measures ANOVA with photoperiod as a between‐subjects factor and apamin dose as a within‐subjects factor, followed by Tukey's multiple comparisons test; ISI and CV comparisons between aCSF and 100 nM apamin within each photoperiod were made with paired *t*‐tests after confirming normality (Shapiro–Wilk test; all W > 0.84, *p* > 0.05). For whole‐cell recordings (Figure 2), tail current amplitude and duration before and after apamin (Figure 2C,E) were analyzed with a mixed‐effects model (restricted maximum likelihood, to accommodate unequal group sizes) with photoperiod as a between‐cell factor and apamin as a within‐cell repeated factor, followed by Šídák's multiple comparisons test; the apamin‐sensitive component (before—after apamin; Figure 2D,F) was compared across photoperiods by ordinary one‐way ANOVA with Tukey's multiple comparisons test. Resting membrane potentials (Supp. Figure 1C) were compared across photoperiods by one‐way ANOVA with Tukey's multiple comparisons test. *Kcnn3* transcript counts (Figure 3) were compared across photoperiods by ordinary one‐way ANOVA. Since we used male and female mice in equal numbers within each cohort, sex differences were analyzed across all cohorts. While no significant sex differences were observed in our dataset, the sample size was too small to draw firm conclusions. Sample sizes were not determined by a priori power calculation; they were chosen to match our previous photoperiod studies of dorsal raphe serotonin neurons, in which comparable effect sizes were detected (Giannoni‐Guzmán et al. 2021; Green et al. 2015). Experimenters were not blinded to photoperiod during slice preparation and recording because housing conditions were known; spike sorting and tail current measurement used fixed criteria applied identically to all groups, and RNAscope image acquisition and quantification were performed blind to photoperiod group.

**FIGURE 1:**
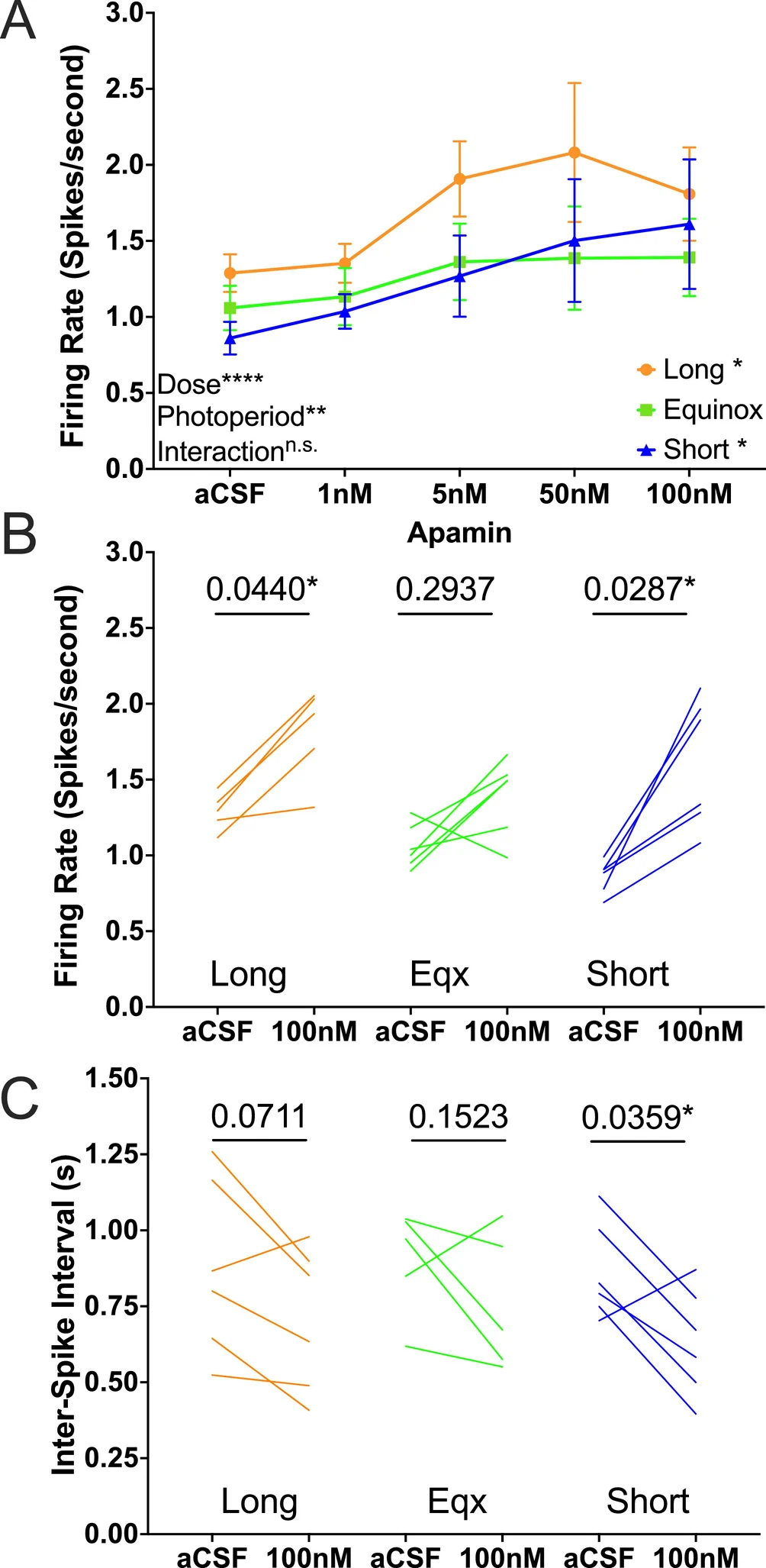
Effects of photoperiod and apamin on firing rate in dorsal raphe nucleus (DRN) serotonin neurons. (A) Mean firing rate (in spikes/s) of serotonin neurons in response to increasing concentrations of apamin (0, 1, 5, 50, and 100 nM; 5 min per treatment) under three photoperiod conditions: Long (orange circles) (*n* = 5), Equinox (green squares) (*n* = 6), and Short (blue triangles) (*n* = 6) presented as mean and standard deviation (SD). Repeated‐measures two‐way ANOVA showed significant effects for apamin Dose *(p* < 0.0001****) and photoperiod (*p* = 0.0044**), while the interaction of these variables was not significant (*p* = 0.0953). (B) Paired comparison of firing rates before and after apamin 100 nM for each photoperiod. Tukey's multiple comparisons revealed significant within‐group increases in firing rate for Long (adj *p* = 0.0440*, 0 vs. 100 nM) and Short (adj *p* = 0.0287*, 0 vs. 100 nM) cohorts. (C) Mean interspike intervals (ISI) per slice of serotonin neurons under each photoperiod, comparing aCSF and 100 nM apamin. Data passed the Shapiro–Wilk normality test (all *p* > 0.05); paired *t*‐tests revealed a significant reduction in ISI following apamin exposure in the Short photoperiod group (Short *p* = 0.0359*, Long *p* = 0.0711, Equinox *p* = 0.1523).

**FIGURE 2:**
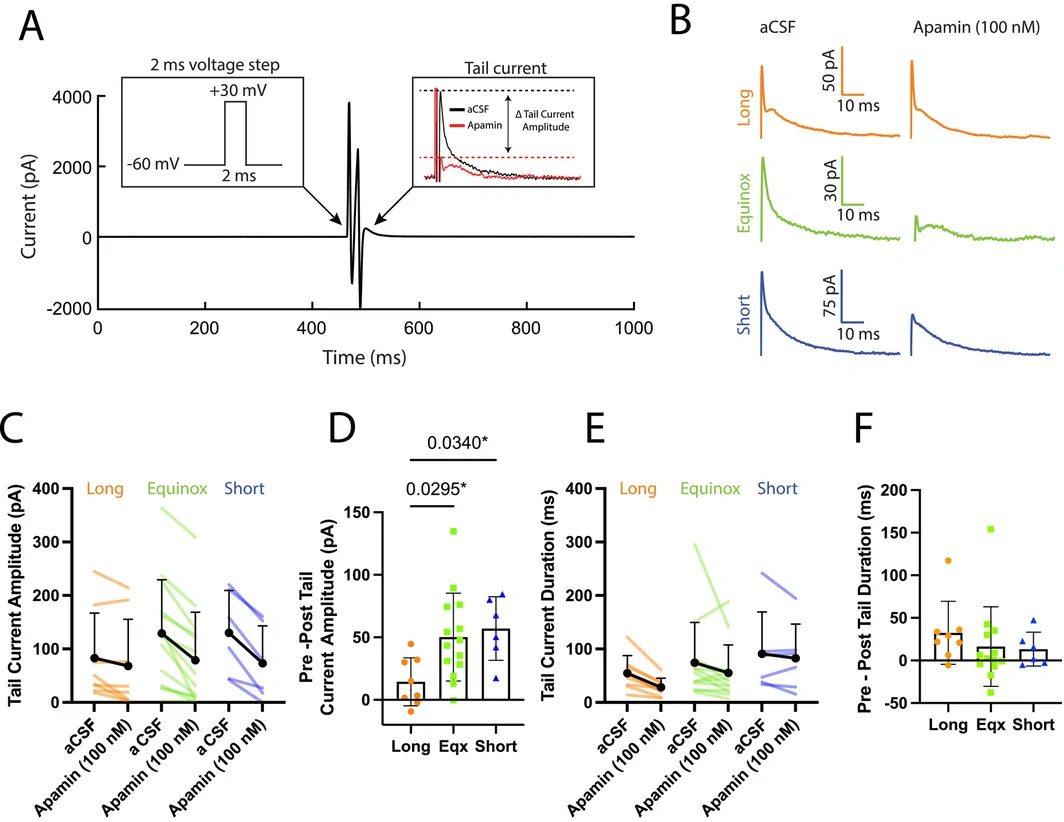
Apamin‐sensitive current amplitude is reduced in Long photoperiod mice. (A) Afterhyperpolarization currents were elicited by applying a 2‐ms pulse to +30 mV from a holding potential of −60 mV, and the resulting tail current following the stimulation artefact was analyzed. (B) Representative traces of afterhyperpolarization currents before and after apamin application for each photoperiod group. (C) Tail current amplitudes before and after apamin treatment. Mixed‐effects model: apamin *F*(1, 25) = 46.68, *p* < 0.0001****; photoperiod *F*(2, 25) = 0.2881, *p* = 0.7521; interaction *F*(2, 25) = 4.796, *p* = 0.0173*. Šídák's multiple comparisons, aCSF versus apamin: Long *p* = 0.4481, Equinox *p* < 0.0001****, Short *p* = 0.0002***. (D) Quantified difference of the peak amplitude tail currents before and after apamin wash. Ordinary one‐way ANOVA, Tukey's multiple comparison test (Long vs. Equinox, *p* = 0.0295; Long vs. Short, *p* = 0.0340; Equinox vs. Short, *p* = 0.8854). Long, *n* = 8 cells, *n* = 5 animals; Equinox, *n* = 14 cells, *n* = 7 animals; Short, *n* = 6 cells, *n* = 5 animals. (E) Tail current durations before and after apamin treatment. Mixed‐effects model: apamin *F*(1, 24) = 6.677, *p* = 0.0163*; photoperiod *F*(2, 24) = 0.8395, *p* = 0.4442; interaction F(2, 24) = 0.5299, *p* = 0.5954. Šídák's multiple comparisons, aCSF vs. apamin: Long *p* = 0.0859, Equinox *p* = 0.3825, Short *p* = 0.8030. (F) Quantified difference of the tail current duration before and after apamin wash. Ordinary one‐way ANOVA, *F*(2, 24) = 0.530, *p* = 0.5954; Tukey's multiple comparison test (Long vs. Equinox, *p* = 0.6458; Long vs. Short, *p* = 0.6501; Equinox vs. Short, *p* = 0.9864). Long, *n* = 8 cells, *n* = 5 animals; Equinox, *n* = 13 cells, *n* = 7 animals; Short, *n* = 6 cells, *n* = 5 animals (one Equinox neuron with no resolvable tail current was excluded from the duration analyses; see Methods).

**FIGURE 3:**
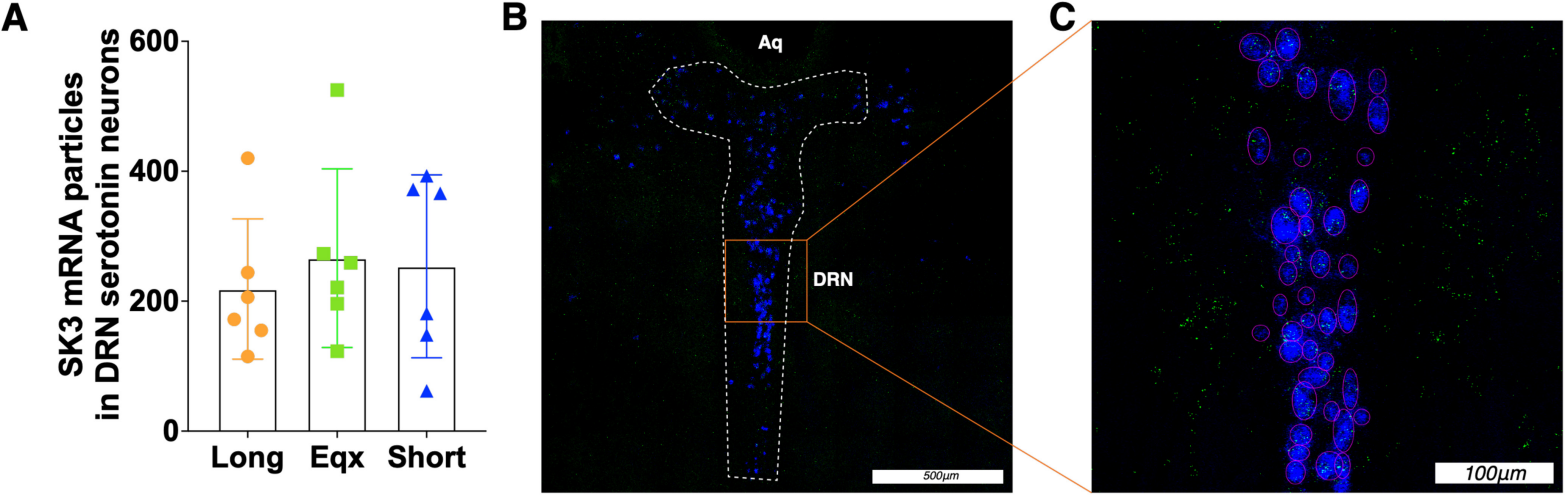
mRNA levels of *Kcnn3* (SK3) in DRN *Tph2*‐positive neurons are similar across photoperiod conditions. (A) Quantification of colocalized *Kcnn3* and *Tph2* positive neurons in the dorsal raphe. Each bar represents the mean number of mRNA particles, with individual data points representing the average value per mouse. The bar represents mean while the error bars indicate standard deviation (SD). Colocalized transcript detection of *Kcnn3* in DRN of C3Hf+/+ mice raised in Long, Equinox or Short photoperiods was similar across photoperiods (*n* = 6 mice per group; one‐way ANOVA, *F*(2, 15) = 0.2164, *p* = 0.8078; Tukey's multiple comparisons test, Long vs. Equinox *p* = 0.8036, Long vs. Short *p* = 0.8873, Equinox vs. Short *p* = 0.9848). (B) Representative image of *Kcnn3* (green) and *Tph2* (blue) signal from Equinox photoperiod. Dashed line outlines the dorsal raphe nucleus; Aq, cerebral aqueduct. (C) Higher magnification view of serotonin neurons in the boxed region in panel B showing the distribution and colocalization of *Kcnn3* fluorescent puncta within *Tph2*‐positive neurons. Magenta ovals represent examples of *Tph2*‐positive clusters where colocalization was quantified. The brightness of *Kcnn3* signaling for panels B and C was increased by 20% in the example shown for visualization purposes.

### 2.7 Use of Artificial Intelligence

During preparation of the revised manuscript, the authors used a generative AI assistant (Claude, Anthropic, Fable 5.1) to help locate and summarize published literature relevant to the reviewers' comments. No AI tool was used to generate, analyze, alter, or interpret experimental data, figures, or statistical results, or to write the manuscript text. All sources identified with AI assistance were retrieved and read by the authors, and all citations were verified against the original publications. The authors take full responsibility for the content of the manuscript.

## 3 Results

### 3.1 Long Photoperiod and SK Channel Inhibition Increase DRN Neuron Activity

To examine the role of SK channels on DRN serotonin firing rate, we inhibited SK channels in a dose‐dependent manner using apamin (0, 1 nM, 5 nM, 50 nM, and 100 nM) and quantified the spontaneous spike rate of serotonin neurons in the presence of 40 μM tryptophan and 3 μM phenylephrine (Methods). Multielectrode array recordings of acute brain slices of the dorsomedial region of the DRN of mice (C3Hf+/+) conceived and raised into adulthood in either Long (summer‐like), Equinox (spring/fall‐like), or Short (winter‐like) photoperiods were used. Serotonin neurons were identified by suppression of spike frequency in response to 8‐OH‐DPAT at the end of each recording, as previously described (Giannoni‐Guzmán et al. 2021; Green et al. 2015).

We observed a significant apamin dose‐dependent increase in the spike rate of Long and Short photoperiod cohorts (*n* = 5–6 per group) (Figure 1A). Repeated measures two‐way ANOVA revealed significant main effects for apamin (F (2.117, 29.64) = 26.16, *p* < 0.0001****) and photoperiod (F (2, 14) = 8.177, *p* = 0.0044**), with a trend toward photoperiod apamin interaction (F (4.234, 29.64) = 2.157 *p* = 0.0953). Tukey's multiple comparison revealed significant within‐group increases in firing rate for Long (adj *p* = 0.0440*) and Short (adj *p* = 0.0287*), but not Equinox (adj *p* = 0.2937), photoperiods following 100 nM apamin exposure (Figure 1B). Additionally, mean interspike intervals (ISIs), which were normally distributed within each photoperiod group (Shapiro–Wilk, all *p* > 0.05), were significantly reduced following 100 nM apamin treatment in the Short photoperiod group only (*p* = 0.0359*), suggesting enhanced apamin effects under short‐day conditions (Figure 1C). The coefficient of variation of ISIs, a measure of spike‐timing regularity, did not change significantly following 100 nM apamin in any photoperiod group (Long, 0.83 ± 0.14 to 0.77 ± 0.10; Equinox, 0.83 ± 0.11 to 0.94 ± 0.24; Short, 0.77 ± 0.04 to 1.11 ± 0.49; mean ± SD per slice, paired t‐tests, all *p* > 0.17), indicating that SK channel inhibition increased firing rate without altering the regularity of spike timing.

### 3.2 Apamin‐Sensitive DRN Tail Currents Are Reduced in Long Photoperiod

To examine potential differences in SK channel currents in mice exposed to different photoperiods, whole‐cell patch‐clamp electrophysiology was performed on neurons within the dorsal raphe nucleus. To identify serotonergic neurons, spontaneous action potentials were recorded in current clamp before and after application of 8‐OH‐DPAT (Supp. Figure 1A), as it has been previously demonstrated that serotonergic cells within the DRN selectively express 5‐HT_1_A receptors, and that these receptors suppress action potential frequency (Assié and Koek 1996; Larsson et al. 1990). Accordingly, only cells that exhibited a 50% or greater reduction in spontaneous action potential frequency in response to 8‐OH‐DPAT were included (Supp. Figure 1B). Resting membrane potentials were not significantly different between the serotonin neurons in the different photoperiod groups (Supp. Figure 1C) (Long = −46.87 ± 3.42 mV; Equinox = −51.74 ± 6.56 mV; Short = −49.63 ± 5.32 mV; mean ± SD; one‐way ANOVA, *F*(2, 24) = 1.912, *p* = 0.170; Tukey's multiple comparisons test: Long vs. Equinox, *p* = 0.1459; Long vs. Short, *p* = 0.6329; Equinox vs. Short *p* = 0.7242). To measure the apamin‐sensitive SK component of AHP tail currents, we simulated single action potentials using a 2 ms depolarization step and then quantified tail current amplitudes before and after bath application of apamin.

Figure 2A shows the voltage protocol and a representative whole‐cell current record. The brief depolarization evokes a large capacitive and action‐current transient, followed, on repolarization to −60 mV, by a slowly decaying outward tail current, which was measured as described in the Methods. Figure 2B shows representative tail currents from one neuron per photoperiod group before and after apamin (100 nM); in Equinox and Short neurons, apamin removed a substantial fraction of the outward tail current, whereas in Long neurons, the apamin‐sensitive fraction was visibly smaller. Apamin reduced tail current amplitude (mixed‐effects model, main effect of apamin, *F*(1, 25) = 46.68, *p* < 0.0001****), and the magnitude of this reduction depended on photoperiod (photoperiod × apamin interaction, *F*(2, 25) = 4.796, *p* = 0.0173*) (Figure 2C); within‐group comparisons showed a significant apamin effect in Equinox (*p* < 0.0001****) and Short (*p* = 0.0002***) but not Long (*p* = 0.4481) neurons. Mean tail current amplitude was 82.33 ± 84.58 pA before and 67.90 ± 87.18 pA after apamin in Long neurons (*n* = 8), 128.7 ± 100.4 and 78.44 ± 89.91 pA in Equinox neurons (*n* = 14), and 129.9 ± 79.29 and 72.80 ± 70.17 pA in Short neurons (*n* = 6). The apamin‐sensitive component (before − after) was significantly smaller in Long (14.4 ± 19.22 pA) than in Equinox (50.3 ± 35.13 pA) or Short (57.1 ± 25.42 pA) neurons (Figure 2D; one‐way ANOVA, *F*(2, 25) = 4.796, *p* = 0.0173; Tukey's multiple comparisons test: Long vs. Equinox *p* = 0.0295, Long vs. Short *p* = 0.0340, Equinox vs. Short *p* = 0.8854). Apamin also shortened tail current duration (mixed‐effects model, main effect of apamin, *F*(1, 24) = 6.677, *p* = 0.0163*), but this effect did not depend on photoperiod (photoperiod *F*(2, 24) = 0.840, *p* = 0.4442; photoperiod × apamin interaction *F*(2, 24) = 0.530, *p* = 0.5954), and within‐group comparisons did not reach significance in any group (Šídák's test: Long *p* = 0.0859, Equinox *p* = 0.3825, Short *p* = 0.8030) (Figure 2E). Mean duration was 73.95 ± 43.07 ms before and 41.55 ± 30.80 ms after apamin in Long neurons (*n* = 8), 81.81 ± 73.69 and 65.41 ± 52.46 ms in Equinox neurons (*n* = 13), and 103.0 ± 73.13 and 89.65 ± 63.32 ms in Short neurons (*n* = 6; mean ± SD). Accordingly, the apamin‐induced change in duration did not differ between photoperiods (Long, 32.4 ± 37.0 ms; Equinox, 16.4 ± 46.6 ms; Short, 13.3 ± 19.9 ms; Figure 2F; one‐way ANOVA, *F*(2, 24) = 0.530, *p* = 0.5954; Tukey's multiple comparisons test: Long vs. Equinox *p* = 0.6458, Long vs. Short *p* = 0.6501, Equinox vs. Short *p* = 0.9864). These results are consistent with the hypothesis that SK currents are reduced in dorsal raphe serotonin neurons in Long photoperiod mice, in line with the previously reported reduced AHP amplitude (Green et al. 2015).

### 3.3 SK3 (*Kcnn3*) Expression in DRN Neurons Is Constant Across Photoperiods

The observed reduction in SK channel currents in DRN serotonin neurons could result from decreased expression of SK channels associated with exposure to Long photoperiods. In the dorsal raphe nucleus, the SK3 channel is the most highly expressed member of the SK channel family (Stocker and Pedarzani 2000). To examine whether the expression levels of the *Kcnn3* (SK3) transcript were altered by environmental photoperiods, we used RNAscope to assess *Kcnn3* specifically in *Tph2‐*expressing serotonergic neurons within the DRN (Figure 3). *Kcnn3* transcripts were detected as discrete fluorescent puncta within *Tph2* ^+^ neurons throughout the dorsal raphe (Figure 3B,C). For each animal, four sections spanning the dorsal raphe (−5.5 to −5.75 mm from Bregma) were imaged, and the number of *Kcnn3* puncta colocalized with *Tph2* signal was quantified with ComDet, yielding a single value per animal. The number of colocalized *Kcnn3* puncta did not differ between photoperiod groups (Long, 218.7 ± 108; Equinox, 266.2 ± 137.5; Short, 253.7 ± 140.9 puncta per animal, mean ± SD; one‐way ANOVA, *F*(2, 15) = 0.2164, *p* = 0.8078; Tukey's multiple comparisons test, all pairwise *p* > 0.05) (Figure 3A). With six animals per group and the observed between‐animal variability (pooled SD ≈ 130 puncta), this comparison had 80% power to detect only a large difference between groups (≈ 230 puncta, or ~95% of the mean; Cohen's *d* = 1.8), so small or moderate changes in *Kcnn3* transcript abundance cannot be excluded. The reduction in the magnitude we previously observed for *Kcnk2* (TREK‐1) transcripts in these neurons under Long photoperiod (~85%–90%; Giannoni‐Guzmán et al. 2021) is comparable to this detection threshold and would therefore likely have been apparent, whereas smaller effects would not have been. These results are consistent with regulation of SK channel function at a posttranscriptional level.

## 4 Discussion

The work presented here reveals that day length, or photoperiod, influences the amplitude of SK channel currents in serotonin dorsal raphe neurons. These findings are consistent with previous work showing increased excitability of serotonin neurons in Long‐photoperiod mice, characterized by lower‐amplitude AHPs compared to serotonin neurons in Short‐photoperiod mice (Green et al. 2015). We have previously also demonstrated photoperiodic transcriptional regulation of TREK‐1 channels, which contribute to setting the resting membrane potential in DRN serotonin neurons (Giannoni‐Guzmán et al. 2021). Here we propose that photoperiodic regulation of DRN serotonin neurons arises from the combined influence of photoperiod on SK and TREK‐1 channels through distinct mechanisms. Unlike Green et al. (2015), we did not detect a significant difference in resting membrane potential between photoperiod groups (Supp. Figure 1C), although Long neurons were again the most depolarized. The absolute potentials we recorded were also more depolarized than in that study, which we attribute to the continuous presence of phenylephrine (3 μM) in our bath, the absence of synaptic blockers, and sampling of membrane potential at break‐in. Given these differences and the smaller sample (6–14 cells per group), the two datasets are not directly comparable, and the absence of a significant resting potential difference here should be regarded as underpowered rather than contradictory.

While *Kcnk2* (TREK‐1) mRNA is markedly reduced (~85%–90%) in DRN serotonin neurons from Long‐photoperiod mice, providing a transcriptional mechanism for their depolarized resting potential (Giannoni‐Guzmán et al. 2021), we found no difference in *Kcnn3* (SK3) transcript levels across photoperiods (Figure 3). This comparison was powered to detect large changes in transcript abundance (see Results), so smaller reductions in *Kcnn3* mRNA cannot be excluded. Nevertheless, a transcriptional change of the size seen for *Kcnk2* would likely have been detected, and the Long‐photoperiod group was numerically only ~18% lower than Equinox, below our detectable threshold. Reduced transcription is therefore unlikely to be the primary factor driving the ~70% reduction in apamin‐sensitive current observed in Long‐photoperiod neurons (Figure 2D). Instead, our data are more consistent with posttranscriptional mechanisms that alter SK channel expression or properties without changing mRNA abundance, such as altered phosphorylation, trafficking, or subunit composition.

SK channel function can be tuned without altering transcript abundance. Native SK channels are multiprotein complexes in which casein kinase 2 (CK2) and protein phosphatase 2A (PP2A) are constitutively associated with the channel; CK2 phosphorylation of channel‐bound calmodulin lowers the apparent Ca^2+^ sensitivity of gating and accelerates deactivation, whereas PP2A reverses these effects (Allen et al. 2007; Bildl et al. 2004), and neurotransmitter signaling can engage this mechanism to suppress SK currents and increase excitability (Maingret et al. 2008). Independently, phosphorylation of the SK2 C‐terminus by protein kinase A (PKA) reduces surface expression of the channel by promoting its internalization (Lin et al. 2008; Ren et al. 2006). Either a reduction in Ca^2+^ sensitivity or a reduction in the number of channels at the membrane would reduce apamin‐sensitive current amplitude, as we observed in Long photoperiod neurons, without altering *Kcnn3* mRNA (Figure 3). Our findings do not distinguish between these possibilities, and we regard posttranscriptional regulation as a hypothesis to be tested directly in the future by assessing SK3 protein and surface expression or the Ca^2+^ dependence of the current across photoperiods. A reduction in the number of functional channels, or in their Ca^2+^ sensitivity, is expected to scale the amplitude of the apamin‐sensitive current, whereas its time course is set largely by the kinetics of the intracellular Ca^2+^ transient and its clearance (Adelman et al. 2012). Therefore, a reduced amplitude without a photoperiod‐dependent change in duration is consistent with fewer or less Ca^2+^ sensitive SK channels responding to an unchanged Ca^2+^ signal, rather than with altered Ca^2+^ handling.

Our results are also consistent with previous work on SK currents and their relation to social and affective behaviors. In a chronic social isolation model, upregulated SK3 currents reduce the excitability of dorsal raphe serotonin neurons, an effect reversed by apamin (Sargin et al. 2016). Although the direction of the SK change differs between the two models, chronic isolation increased SK3‐mediated currents and reduced serotonin neuron activity, whereas Long photoperiod reduced apamin‐sensitive current and increased activity; both point to SK channels as a bidirectional lever through which environmental history sets serotonin neuron excitability. Notably, the isolation‐induced increase in SK current was accompanied by increased SK3 expression (Sargin et al. 2016), whereas the photoperiod‐induced decrease occurred without a change in *Kcnn3* mRNA levels, suggesting that different environmental signals may act on SK channels at distinct regulatory levels. Chronic isolation has been shown to increase anxiety/depression‐like behaviors (Dang et al. 2015; Fone and Porkess 2008; Koike et al. 2009; Lukkes et al. 2009; Shimizu et al. 2016; Wallace et al. 2009). Systemic administration of apamin reduced anxiety/depression‐like behaviors in mice under chronic isolation (Galeotti et al. 1999; Sargin et al. 2016; van der Staay et al. 1999), and SK3 null mice show enhanced hippocampal serotonin release and antidepressant‐like behavioral phenotypes (Jacobsen et al. 2008). These findings underscore the critical role of SK channels as dynamic modulators of serotonin neuron excitability, enabling neurons to rapidly adjust their firing properties in response to environmental cues such as photoperiod changes and social stress. By fine‐tuning afterhyperpolarization, SK channels serve as a key mechanism through which external factors can shape mood and anxiety‐related behaviors.

Differential expression of SK1, SK2, and SK3 can change the biophysical properties (such as conductance and Ca^2+^ sensitivity) of the SK current (Sailer et al. 2002). In the dorsal raphe nucleus, SK3 is most highly expressed (Stocker and Pedarzani 2000). Our study focused on SK3 expression in *Tph2‐*positive neurons within the DRN, providing a targeted view of SK channel regulation. It is possible that shifts in the relative expression of SK1 and SK2 in response to photoperiod could alter the excitability of serotonergic neurons. Examining changes in SK channel composition could reveal adaptive mechanisms underlying the regulation of neuronal excitability and mood.

A limitation of the present study is that multielectrode array recordings were conducted without fast synaptic blockers. Given that serotonergic neurons in the dorsal raphe receive robust excitatory and inhibitory synaptic input (Rood et al. 2014), we cannot rule out that apamin‐induced changes in synaptic transmission—such as spontaneous IPSPs or EPSPs—may have contributed to the observed alterations in firing rate. However, our primary goal was to assess the overall effect of SK channel blockade on the spontaneous activity of 5‐HT neurons under physiologically relevant conditions. By preserving endogenous synaptic inputs, we aimed to capture the net effect of SK modulation within intact local circuitry. While the results likely reflect a combination of intrinsic and network‐level effects, the use of apamin provides direct pharmacological insight into the functional contribution of SK channels to 5‐HT neuron excitability. Future experiments incorporating synaptic blockers or cell‐type specific approaches could help further isolate the intrinsic role of SK channels in these neurons.

One consideration when interpreting our pharmacology is that α1‐adrenergic signaling can modulate SK channels. In rat dorsal raphe neurons, α1 agonists augment an apamin‐sensitive late afterhyperpolarization by mobilizing Ca^2+^ from intracellular stores (Pan et al. 1994), whereas in mouse dorsal raphe serotonin neurons, phenylephrine (10 μM) partially inhibits the SK‐mediated afterhyperpolarization current, with apamin producing a substantially larger block (Wang et al. 2022); neurotransmitter inhibition of SK channels can occur through CK2‐dependent phosphorylation of channel‐bound calmodulin that lowers Ca^2+^ sensitivity (Maingret et al. 2008). In the present study, phenylephrine was held constant at 3 μM across photoperiod groups and between baseline and apamin conditions, so it cannot account for the between‐group difference in apamin‐sensitive current, although partial occlusion of the SK current by phenylephrine could lead us to underestimate the total SK current in all groups. Photoperiod also programs the firing‐rate response of these neurons to phenylephrine, without a detectable change in α1 receptor expression or phenylephrine potency (Green et al. 2015), an effect attributed to increased intrinsic excitability. Therefore, we cannot fully rule out an interaction between α1 signaling and SK function that varies with photoperiod, although the unchanged receptor pharmacology argues against a difference in the α1 pathway itself. Further research could answer this by measuring the photoperiod dependence of tail currents in the presence of an α1 antagonist.

All tissue was collected, and all recordings were made during the light phase, at the midpoint of the light period in every photoperiod, so that groups were compared at a fixed phase relative to the light cycle and consistent with our previous photoperiod studies of these neurons (Giannoni‐Guzmán et al. 2021; Green et al. 2015). This choice has limitations. Serotonin neuron firing is higher during the dark (active) phase, and locus coeruleus neuron activity, which provides the noradrenergic drive to the dorsal raphe, also varies between the light and dark phases (Zhang et al. 2026). Whether SK channel expression or function in dorsal raphe serotonin neurons varies over the course of the day is not known. Daily rhythms in *Tph2* expression have been reported in the raphe (Malek et al. 2005, 2007), but, to our knowledge, *Kcnn3* has not been examined. In the suprachiasmatic nucleus, the apamin‐sensitive afterhyperpolarization does not differ between day and night (Teshima et al. 2003). Examining SK currents during the dark phase would be an important extension of this work.

Male and female mice were used in equal numbers in each cohort, and no significant effect of sex was detected. However, the cohorts were not powered for this comparison, and females were not staged for estrous cycle. A recent study reported higher spontaneous firing and a more depolarized resting membrane potential in dorsal raphe serotonin neurons of female, relative to male C57BL/6 J mice, dependent on estrogen receptor α signaling, and observed in females at diestrus (Torres Irizarry et al. 2024). Those recordings were made in a melatonin‐deficient strain without α1‐adrenergic stimulation and are not directly comparable to ours, but they raise the possibility that melatonin‐dependent photoperiodic programming interacts with gonadal hormone state. More generally, two of our datasets are modest in size: the whole‐cell recordings (6–14 cells per group) and the RNAscope quantification (6 animals per group), in which between‐animal variability limited sensitivity to large effects.

Our findings open several avenues for further investigation into the molecular and functional aspects of SK channel regulation by photoperiod in serotonin neurons. Future studies should aim to pinpoint the precise posttranscriptional mechanisms—such as phosphorylation events, trafficking dynamics, or changes in subunit assembly—that drive the photoperiod‐dependent modulation of SK currents. In addition, exploring how these regulatory processes vary across different developmental stages may reveal critical windows during which serotonergic circuits are most sensitive to environmental cues.

The present study contributes significantly to our understanding of how environmental factors, such as seasonal changes in day length, can shape neural function at the cellular level. By demonstrating that photoperiod influences SK channel activity in serotonin neurons, these results contribute to the growing mechanistic framework linking external environmental cues to intrinsic neuronal excitability and behavior. This understanding may have significant implications for developing targeted therapies for mood disorders and other conditions associated with dysregulated serotonin signaling. Positive modulators of SK channels such as NS309 or CyPPA, which increase the Ca^2+^ sensitivity of the channel, would provide a direct test of whether restoring SK current in Long‐photoperiod neurons normalizes their excitability (Hougaard et al. 2007; Strøbæk et al. 2004). Interventions targeting SK channel function could in the future offer a novel approach to alleviating symptoms of depression and anxiety, highlighting the translational potential of this research for neuropsychiatric treatments.

## Author Contributions

**Manuel A. Giannoni‐Guzmán:** conceptualization, investigation, writing – original draft, writing – review and editing, data curation, methodology, visualization, validation, formal analysis. **José C. Zepeda:** investigation, visualization, methodology, writing – review and editing, data curation, writing – original draft. **Anna Kamitakahara:** investigation, methodology. **Valerie Magalong:** investigation, methodology. **Pat Levitt:** funding acquisition, supervision, resources. **Brad A. Grueter:** resources, supervision, project administration, validation, writing – review and editing, funding acquisition. **Douglas G. McMahon:** conceptualization, funding acquisition, writing – review and editing, writing – original draft, supervision, resources.

## Funding

This research was supported by the National Science Foundation Graduate Research Fellowship Program (NSF GRFP 1937963 to J.C.Z), the National Institutes of Health (Grants NIH R01 MH108562 to D.G.M. and B.A.G.), and the Postdoctoral Program in Functional Neurogenomics (Grant 5T32MH065215‐13).

## Conflicts of Interest

The authors declare no conflicts of interest.

## Supporting information


**Figure S1:** Identification of 8‐OH‐DPAT‐sensitive serotonergic neurons by suppression of spontaneous firing. (A) Representative current‐clamp recordings of spontaneous action potentials before and after bath application of 8‐OH‐DPAT (5 μM) from an 8‐OH‐DPAT‐sensitive neuron (top) and an 8‐OH‐DPAT‐insensitive neuron (bottom). (B) Spontaneous action potential frequency (Hz) before and after 8‐OH‐DPAT for each neuron included in the study, by photoperiod group (Long, *n* = 9; Equinox, *n* = 13; Short, *n* = 6 cells); lines connect values from the same neuron. Only neurons whose firing rate fell by ≥ 50% in 8‐OH‐DPAT were classified as serotonergic and included. (C) Resting membrane potential measured immediately after break‐in (Long, *n* = 8; Equinox, *n* = 13; Short, *n* = 6 cells; mean ± SD). One‐way ANOVA, *F*(2, 24) = 1.912, *p* = 0.170; Tukey's multiple comparisons test: Long versus Equinox, *p* = 0.1459; Long vs. Short, *p* = 0.6329; Equinox vs. Short, *p* = 0.7242.

## Data Availability

The data that support the findings of this study (per‐slice multielectrode array firing rates, interspike intervals and coefficients of variation; per‐cell tail current amplitudes and durations; per‐animal RNAscope puncta counts; and the GraphPad Prism files used to generate all figures) and the Python script used to compute interspike intervals are openly available in Zenodo at 10.5281/zenodo.23478977. Raw electrophysiology recordings are available from the corresponding author on reasonable request.
