# Supplementary material for: Photoperiodic Regulation of SK Channels in Dorsal Raphe Serotonin Neurons
Source: Eur J Neurosci. 2026 Nov 5;64(9):e70752. doi: 10.1111/ejn.70752 (PMC13735882; doi:10.1111/ejn.70752)
Supplement: Supplementary file 1 [file EJN-64-0-s001.pdf]

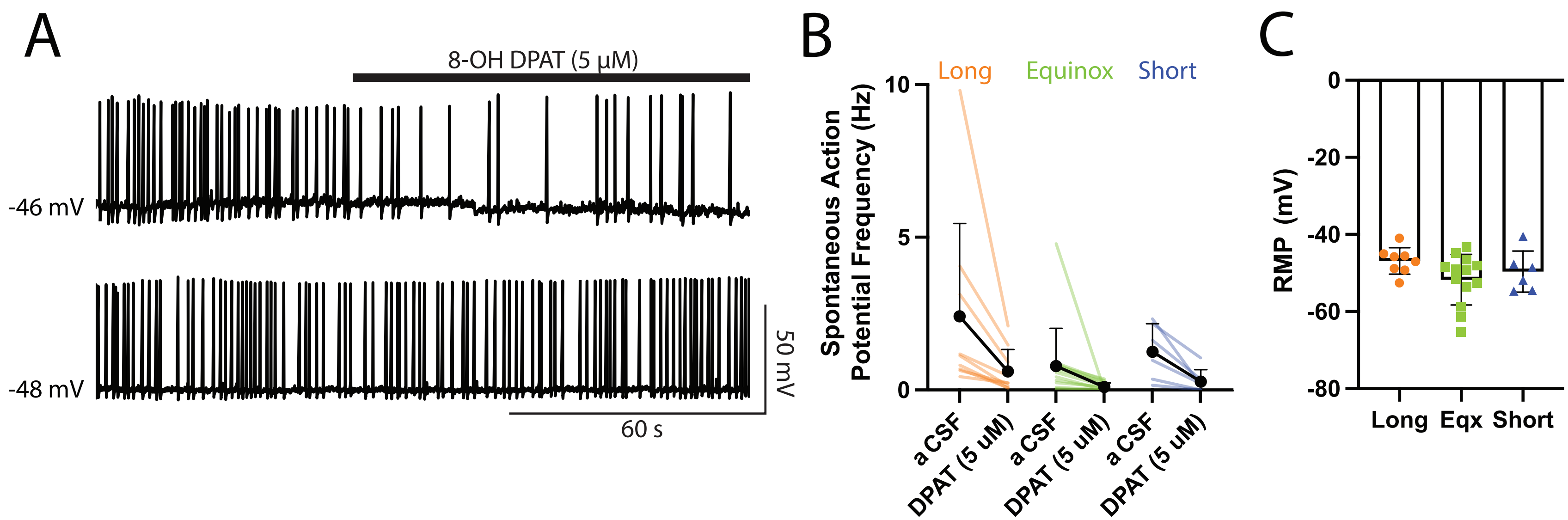

Supplementary Figure 1. Identification of 8-OH-DPAT-sensitive serotonergic neurons by suppression of spontaneous firing. A) Representative current-clamp recordings of spontaneous action potentials before and after bath application of 8-OH-DPAT (5 µM) from an 8-OH-DPAT-sensitive neuron (top) and an 8-OH-DPAT-insensitive neuron (bottom). **B)** Spontaneous action potential frequency (Hz) before and after 8-OH-DPAT for each neuron included in the study, by photoperiod group (Long, n = 9; Equinox, n = 13; Short, n = 6 cells); lines connect values from the same neuron. Only neurons whose firing rate fell by ≥50% in 8-OH-DPAT were classified as serotonergic and included. **C)** Resting membrane potential measured immediately after break-in (Long, n = 8; Equinox, n = 13; Short, n = 6 cells; mean ± SD). One-way ANOVA, $F(2, 24) = 1.912$, $p = 0.170$; Tukey's multiple comparisons test: Long vs Equinox, $p = 0.1459$; Long vs Short, $p = 0.6329$; Equinox vs Short, $p = 0.7242$.

.
